# Chronic Exposure to *Gelsemium* Preparations Alters Mitochondrial Bioenergetics, Neurite Outgrowth, and Akt/mTOR Signaling in Human Neuronal Cells

**DOI:** 10.3390/ijms27125409

**Published:** 2026-06-16

**Authors:** Imane Lejri, Amandine Grimm, Pascal Trempat, Stephanie Chanut, Naoual Boujedaini, Anne Eckert

**Affiliations:** 1 Neurobiology Laboratory for Brain Aging and Mental Health, Transfaculty Research Platform, Molecular & Cognitive Neuroscience, University of Basel, CH-4002 Basel, Switzerland; imane.lejri@upk.ch (I.L.); amandine.grimm@unibas.ch (A.G.); 2 Neurobiology Lab for Brain Aging and Mental Health, Psychiatric University Clinic, Wilhelm Klein-Str. 27, CH-4002 Basel, Switzerland; 3 Laboratoire Boiron, 2 Avenue de l’Ouest Lyonnais, 69510 Messimy, France; pascal.trempat@boiron.fr (P.T.); stephanie.chanut@boiron.fr (S.C.); 4 Département de Recherche Clinique, Institut de Cancérologie de Lorraine–ICL, 6 Avenue de Bourgogne, 54519 Vandœuvre-lès-Nancy Cedex, France; naoual.boujedaini@gmail.com

**Keywords:** *Gelsemium* preparations, mitochondrial bioenergetics, neurite outgrowth, neuroprotection, Akt/mTOR signaling

## Abstract

Stress-related psychiatric disorders are frequently associated with impaired mitochondrial function, altered neuronal energy metabolism, and reduced neuroplasticity. Intracellular pathways such as PI3K/Akt and mTOR play central roles in regulating mitochondrial bioenergetics and neuronal structural adaptation. *Gelsemium* is traditionally used in integrative and homeopathic practice; however, the cellular effects of prolonged exposure to high serial dilutions remain insufficiently characterized. This study aimed to examine the effects of chronic exposure to *Gelsemium* preparations on mitochondrial function and neuronal plasticity in vitro. Human SH-SY5Y neuroblastoma cells were treated for 14 days with different *Gelsemium* preparations 9C, 15C, 30C. Mitochondrial bioenergetics, reactive oxygen species (ROS) production, cell viability, neurite outgrowth, and phosphorylation of Akt and mTOR were assessed using complementary biochemical, imaging, and signaling analyses. Chronic exposure to *Gelsemium* preparations was associated with increased ATP production, increased mitochondrial respiration and glycolytic activity, reduced oxidative stress, improved cell viability, and increased neurite outgrowth compared with untreated controls. These changes were accompanied by increased phosphorylation of Akt and mTOR. The convergence of bioenergetic, redox, morphological, and signaling readouts suggests a coordinated cellular response under prolonged exposure conditions. These findings indicate that chronic exposure to *Gelsemium* preparations (9C, 15C, 30C) is associated with coordinated changes in mitochondrial bioenergetics, redox balance, and Akt/mTOR signaling in neuronal cells under in vitro conditions.

## 1. Introduction

Stress-related mental disorders such as anxiety and depression represent a significant and growing global health concern and are frequently associated with alterations in neuronal energy metabolism and reduced neuroplasticity [1,2]. Increasing evidence points to mitochondrial dysfunction as a pivotal factor in stress-related neuronal alterations, characterized by impaired ATP production, increased reactive oxygen species (ROS), and compromised cellular resilience and neuroplasticity [2,3,4]. Both clinical observations and experimental models have highlighted a close relationship between mitochondrial bioenergetics, oxidative stress, and neuronal adaptation to stress [5,6,7,8]. Recent research highlights a bidirectional link between anxiety and brain mitochondrial function, with mitochondrial and metabolic alterations observed in both anxious individuals and patients with mitochondrial disorders [9]. Targeting mitochondrial function has been proposed as a potential strategy for alleviating stress-associated symptoms [5,9,10,11]. Mitochondria are vital for ATP synthesis via oxidative phosphorylation, regulation of oxidative stress, and initiation of cell survival signaling, all of which are fundamental to maintaining neuronal function and resilience under stress [12,13]. Mitochondrial bioenergetics and dynamics are modulated by intracellular pathways, including the phosphatidylinositol 3-kinase (PI3K)/Akt and mammalian target of rapamycin (mTOR) signaling cascades. Phosphorylation of Akt enhances mitochondrial function and promotes neuronal survival, while mTOR regulates protein synthesis essential for neurite outgrowth and synaptic remodeling [14,15,16]. Dysregulation of these pathways contributes to anxiety-related neuronal deficits, suggesting potential targets for therapeutic intervention [17,18]. Although significant progress has been made in the study of psychiatric disorders such as anxiety and depression, their pathogenesis remains incompletely understood. Moreover, the clinical management of these conditions continues to face major challenges, including serious adverse effects and resistance to antipsychotic medications. Emerging evidence highlights mTOR as a key signaling hub involved in neuronal growth, synaptogenesis, and synaptic plasticity [18,19]. In particular, the PI3K-AKT/mTOR pathway has been identified as a critical mediator of the rapid antidepressant effects observed in both clinical and preclinical studies [18]. Dysregulation of this signaling cascade has been strongly implicated in the pathogenesis of several neurodevelopmental and psychiatric disorders [18].

*Gelsemium* is traditionally used in integrative and homeopathic practice and has been investigated in several experimental studies for its effects on neuronal systems. Previous experimental studies have reported that *Gelsemium* modulates behavioral responses to stress, neurosteroid production, and neuronal gene expression, including pathways related to calcium signaling and inflammation, in both animal and neuronal cell models [20,21,22,23,24]. Extending these observations, our previous work demonstrated that *Gelsemium* 3C and 5C influence mitochondrial activity and neurite outgrowth in neuronal cells [25]. More recently, we further investigated their effects under serum deprivation–induced cellular stress and showed improvements in mitochondrial bioenergetics, redox balance, and neurite outgrowth [13], suggesting a role for mitochondrial modulation in stress-related neuronal adaptation. However, the cellular effects of prolonged exposure at different dilutions remain insufficiently characterized, particularly regarding to mitochondrial bioenergetics and signaling pathways involved in neuronal plasticity.

In clinical homeopathic practice, *Gelsemium* 30C is marketed for anxiety-related complaints and is administered as repeated oral doses over several days [26]. This chronic treatment paradigm was selected to reflect the repeated dosing regimens described for *Gelsemium* preparations in clinical homeopathic practice.

Accordingly, Hahnemannian centesimal dilutions of *Gelsemium* (9C, 15C, and 30C), where “C” denotes the centesimal dilution scale (×100), were investigated. For methodological accuracy, these are referred to here as homeopathic dilutions; elsewhere in the manuscript, the term “*Gelsemium* preparations” is used.

In the present study, a 14-day treatment period was implemented to examine whether chronic exposure to *Gelsemium* preparations (9C, 15C, 30C) induces measurable changes in mitochondrial function, oxidative stress, cell viability, neurite outgrowth, and Akt/mTOR signaling in human SH-SY5Y neuronal cells. By combining bioenergetic profiling, morphological analysis, and signaling pathway assessment, this work aims to characterize cellular responses to prolonged exposure under controlled in vitro conditions.

## 2. Results

The effect of a “chronic” treatment of 2 weeks with the different *Gelsemium* preparations 9C, 15C, and 30C was assessed for their ability to modulate mitochondrial function in SH-SY5Y cells. Nerve Growth Factor (NGF, 50 ng/mL), a known promoter of cell growth and survival, served as a positive control. Since vehicle treatment had no measurable effect in any assay and did not differ significantly from medium-treated control cells (all *p* > 0.05; Supplementary Figures S1 and S2), the effects of *Gelsemium* preparations were compared to the control condition (CTRL; cells treated with medium alone) in all subsequent experiments.

### 2.1. Chronic Treatment with Gelsemium Preparations Improves ATP Production in SH-SY5Y Cells

**Figure 1:**
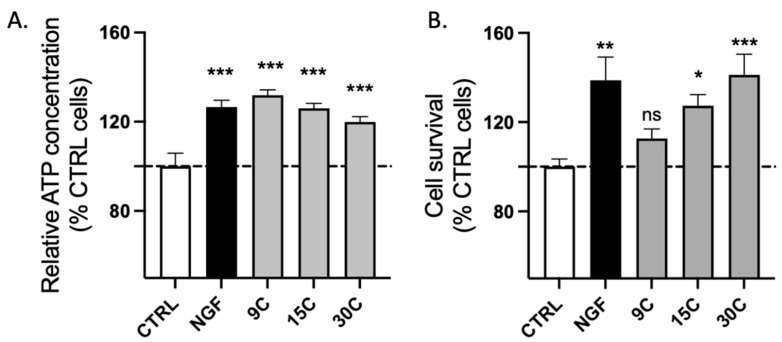
Effect of chronic treatment for 14 days with *Gelsemium* preparations (9C, 15C, and 30C) or nerve growth factor (NGF) on (**A**) ATP production and (**B**) cell viability (Table 1). Data represent mean ± SEM from three independent biological experiments performed using different cell passages and on different experimental dates. The reported *n* values (ATP assay: *n* = 17–29 replicates per condition; MTT assay: *n* = 17–33 replicates per condition) correspond to the total number of technical replicates pooled across the three independent experiments. Values were normalized to 100% of untreated control cells (CTRL, dashed line). Statistics: One-way ANOVA (*p* < 0.001: (**A**,**B**)) with post hoc Dunnett’s multiple comparisons tests. For (**A**), 9C/15C/30C vs. CTRL: *** *p* < 0.001; NGF vs. CTRL: *** *p* < 0.01; 9C/15C/30C vs. NGF: not significant. For (**B**), 9C/15C/30C vs. CTRL: not significant (*p* = 0.51), * *p* < 0.05, *** *p* < 0.001; NGF vs. CTRL: ** *p* < 0.01; 9C/15C/30C vs. NGF: not significant.

First, the effect of a 2-week chronic treatment with *Gelsemium* preparations on ATP production in SH-SY5Y cells was evaluated (Figure 1A). The 9C, 15C, and 30C preparations significantly increased ATP levels by approximately +31.9%, +26.1%, and +19.9%, respectively, compared to CTRL cells. Chronic treatment with NGF also significantly increased ATP production by +26.6% relative to CTRL. Yet this effect remained lower than that induced by *Gelsemium* 9C and comparable to that observed with *Gelsemium* 15C (Figure 1A).

### 2.2. Neurotrophic Effects of Chronic Gelsemium Treatment on SH-SY5Y Cell Viability

To further investigate the neurotrophic effects of *Gelsemium* preparations, a 2-week chronic treatment was applied to SH-SY5Y neuroblastoma cells, and cell viability was assessed using the MTT assay (Figure 1B). In contrast to ATP production, higher *Gelsemium* preparations were more effective in enhancing MTT activity. Specifically, NGF and *Gelsemium* 15C and 30C significantly increased MTT values, with the highest homeopathic dilution 30C exerting the strongest effect: +38.7%, +27.4%, and +41.2%, respectively, compared to CTRL cells (Figure 1B). Chronic treatment with *Gelsemium* 9C slightly increased +12.6% the metabolic activity of SH-SY5Y cells, although this effect did not reach statistical significance (*p* = 0.51; Figure 1B).

### 2.3. Chronic Gelsemium Preparations Exposure Lowers Total Superoxide Anion Production

**Figure 2:**
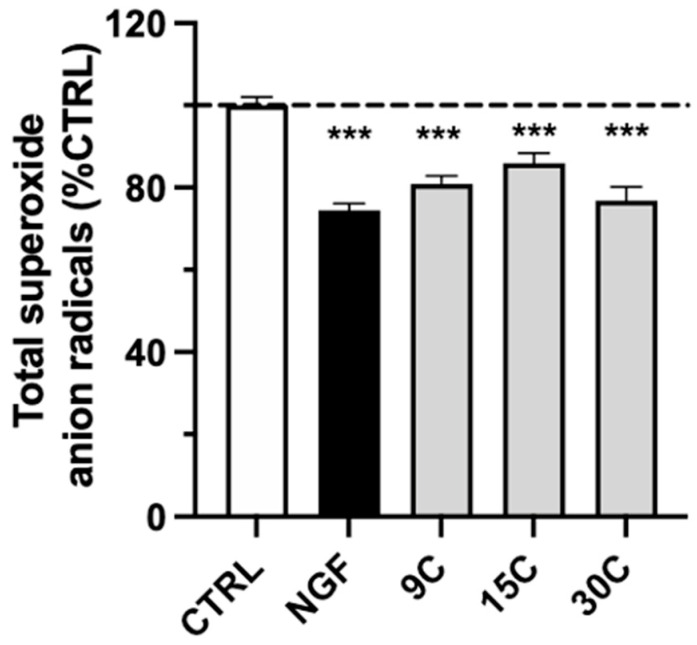
Reduction in total superoxide anion radical levels after 2 weeks of chronic treatment with *Gelsemium* preparations (Table 2). Values represent mean ± SEM from three independent biological experiments performed using different cell passages and on different experimental dates. The reported *n* values (*n* = 14–18 replicates per condition) correspond to the total number of technical replicates pooled across the three independent experiments and are normalized to 100% of control cells treated with medium alone (CTRL, dashed line). Statistics: One-way ANOVA (*p* < 0.001) with post hoc Dunnett’s multiple comparisons tests: 9C/15C/30C vs. CTRL: *** *p* < 0.001; NGF vs. CTRL: *** *p* < 0.001; 9C/15C/30C vs. NGF: not significant.

To evaluate the impact of chronic *Gelsemium* preparations on oxidative stress, total superoxide anion radicals were quantified using the DHE probe. DHE staining showed a marked decrease in total superoxide levels for all *Gelsemium* preparations and NGF compared to CTRL cells (Figure 2). Total superoxide anion levels were significantly reduced following chronic *Gelsemium* preparation treatments, with decreases of −19.1%, −14.2%, and −23.1% for the 9C, 15C, and 30C dilutions, respectively. NGF chronic treatment decreased total superoxide anion levels by −25.6% compared to the CTRL condition (Figure 2).

### 2.4. Chronic Gelsemium Preparations Exposure to Increases Mitochondrial and Glycolytic Activity

**Figure 3:**
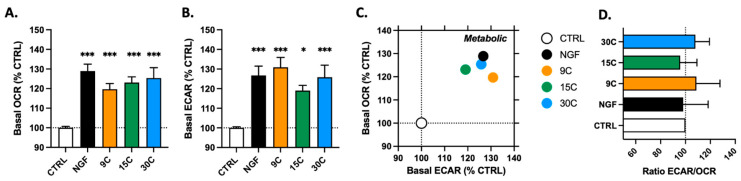
Modulation of mitochondrial respiration and glycolysis by chronic exposure to *Gelsemium* preparations (Table 3). SH-SY5Y cells were treated for 2 weeks with high-dilution *Gelsemium* preparations. Basal oxygen consumption rate (OCR, panel (**A**)) and basal extracellular acidification rate (ECAR, panel (**B**)) were measured simultaneously using the Seahorse XF HS Mini Analyzer (Agilent, Santa Clara, CA, USA). Panel (**C**) shows the bioenergetic phenotype profile determined by plotting extracellular acidification rate (ECAR) against oxygen consumption rate (OCR), normalized to CTRL (100%, dashed lines). Chronic exposure to *Gelsemium* preparations was associated with increased OCR and ECAR values, consistent with an overall enhancement of cellular bioenergetic activity. Data represent mean ± SEM from 12 independent biological experiments performed using different SH-SY5Y cell passages and on different experimental dates. The reported *n* values (OCR: *n* = 40–48 replicates per condition; ECAR: *n* = 37–48 replicates per condition) correspond to the total number of technical replicates pooled across the 12 independent biological experiments and are normalized to 100% of control cells treated with medium alone (CTRL, dashed lines). Statistics: One-way ANOVA (*p* < 0.001: (**A**,**B**)) with post hoc Dunnett’s multiple comparisons tests. For (**A**): 9C/15C/30C vs. CTRL: *** *p* < 0.001; NGF vs. CTRL *** *p* < 0.001; 9C/15C/30C vs. NGF: not significant. For (**B**): 9C/15C/30C vs. CTRL * *p* < 0.05, *** *p* < 0.001; NGF vs. CTRL *** *p* < 0.001; 9C/15C/30C vs. NGF: not significant. Panel (**D**) shows the ECAR/OCR ratio normalized to CTRL (%, dashed line) calculated from 12 independent biological experiments. Individual data points represent biological replicates and bars indicate mean values. Statistical analysis was performed using a mixed-effects model (REML) followed by Dunnett’s multiple comparisons test versus CTRL. A significant overall treatment effect was observed (F (2.311, 23.11) = 3.716, *p* = 0.03); however, no individual treatment group differed significantly from CTRL after correction for multiple comparisons.

Mitochondrial oxidative phosphorylation (OXPHOS) and cellular glycolysis are the two main pathways responsible for ATP production. To assess whether *Gelsemium* preparations 9C, 15C, and 30C modulate one or both of these metabolic pathways, we evaluated their effects following a 2-week chronic treatment in SH-SY5Y cells (Figure 3). Oxygen consumption rate (OCR), an indicator of basal mitochondrial respiration, and extracellular acidification rate (ECAR), a proxy for glycolytic activity, were measured simultaneously in real-time.

*Gelsemium* 9C, 15C, and 30C significantly increased OCR levels by +19.7%, +23%, and +25.4%, respectively. The positive control NGF induced an increase in OCR levels up to +29% relative to cells treated with medium alone (CTRL) (Figure 3A). Regarding glycolytic activity, chronic treatment with *Gelsemium* 9C, 15C, and 30C significantly increased ECAR levels by +30.9%, +19.2% and +25.8%, respectively, compared to CTRL cells. NGF improved the ECAR levels by +26.7% compared to CTRL cells (Figure 3B). The bioenergetic phenotype analysis of CTRL cells (Figure 3C, normalized to 100%), representing OCR versus ECAR under the various treatment conditions, revealed that *Gelsemium* 9C, 15C, and 30C enhanced both mitochondrial respiration and glycolytic activity. These changes are consistent with an overall increase in cellular bioenergetic activity. To further quantify the bioenergetic phenotype, ECAR/OCR ratios were calculated for each independent experiment and statistically compared between groups (Figure 3D). While a significant overall treatment effect was detected using a mixed-effects model (REML) (*p* = 0.03), no individual treatment group differed significantly from CTRL after correction for multiple comparisons.

### 2.5. Chronic Gelsemium Preparations Exposure Stimulates Neurite Network Complexity

Increased OCR and ECAR reflect enhanced bioenergetics, which may support the energy-intensive processes of neurite growth and synaptic reorganization.

**Figure 4:**
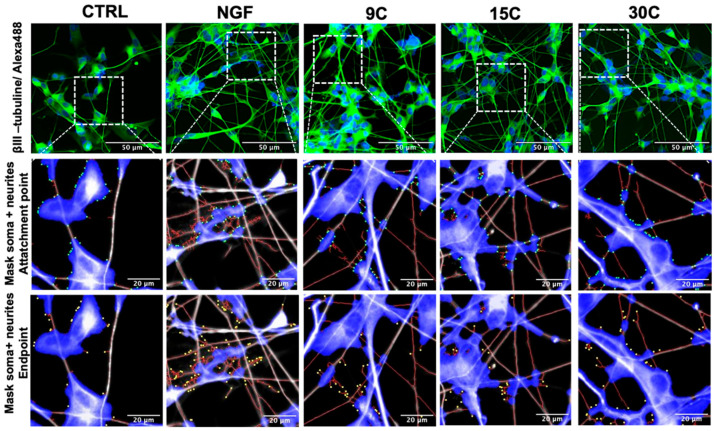
*Gelsemium* preparations enhance neuritogenesis in human neuroblastoma cells following 2 weeks of chronic treatment in 2D culture. Representative images were randomly acquired using the Cytation 3 imaging system at 20× magnification and cropped using NeurophologyJ. Original uncropped images corresponding to these representative fields are shown in Supplementary Figure S3. Cells were immunostained with βIII-tubulin/Alexa Fluor 488 (green) to visualize neurites and DAPI (blue) to label nuclei. Upper panels show representative neurite networks following chronic treatment with nerve growth factor (NGF) or *Gelsemium* preparations. The middle and lower panels show enlarged segmentation mask views of selected regions illustrating attachment points and endpoints, respectively. Color coding: blue—cell soma; red—neurites; green dots—attachment points; yellow dots—endpoints.

**Figure 5:**
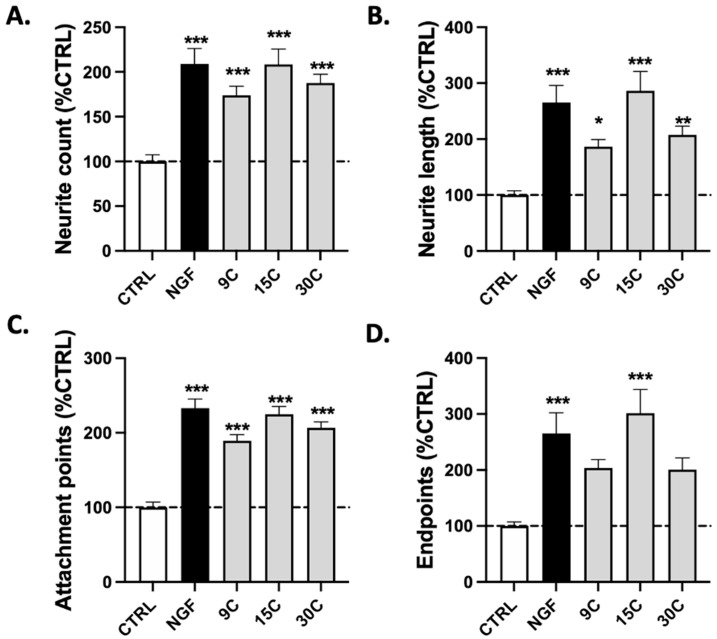
*Gelsemium* preparations enhance neurite outgrowth parameters in neuroblastoma cells after 2 weeks of chronic treatment in 2D culture (Table 4). Quantification of neurite outgrowth from Cytation 3 random images was performed using NeurophologyJ and GraphPad Prism 9 (version 9.3.1). *Gelsemium* 9C, 15C, and 30C significantly increased (**A**) neurite count, (**B**) neurite length, (**C**) number of attachment points, and (**D**) number of endpoints. The effects of *Gelsemium* preparations were of similar magnitude to those observed with NGF, as shown. Values represent mean ± SEM from three independent biological experiments performed using different SH-SY5Y cell passages and on different experimental dates. The *n* values reported in Table 4 correspond to the total number of individual cells analyzed across the three independent biological experiments. Data were normalized to 100% of the control (CTRL, dashed lines) cells treated with medium alone (Table 4). Statistics: One-way ANOVA (*p* < 0.001: (**A**–**D**)) with post hoc Dunnett’s multiple comparisons tests. For (**A**): 9C/15C/30C vs. CTRL *** *p* < 0.001; NGF vs. CTRL *** *p* < 0.001; 9C/15C/30C vs. NGF: not significant. For (**B**): 9C/15C/30C vs. CTRL * *p* < 0.05, ** *p* < 0.01, *** *p* < 0.001; NGF vs. CTRL *** *p* < 0.001; 9C/15C/30C vs. NGF: not significant. For (**C**): 9C/15C/30C vs. CTRL *** *p* < 0.001; NGF vs. CTRL *** *p* < 0.001; 9C/15C/30C vs. NGF: not significant. For (**D**): 9C vs. CTRL *p* = 0.06, *** *p* < 0.001; 30C vs. CTRL *p* = 0.07; NGF vs. CTRL *** *p* < 0.001; 9C/15C/30C vs. NGF: not significant.

To investigate the effect of a chronic treatment of *Gelsemium* on neurite outgrowth, three different dilutions of *Gelsemium* 9C, 15C, and 30C, as well as vehicle, were tested on differentiated SH-SY5Y cells using the 2D cell culture method (Figure 4 and Figure 5, Supplementary Figure S2). NGF (50 ng/mL), a well-established promoter of neuronal growth and survival, was used as a positive control.

Under these conditions, NGF treatment increased neurite count by 109%, total neurite length by 165%, the number of attachment points by 133%, and the number of endpoints by 165% compared with CTRL cells (Figure 4 and Figure 5, Supplementary Figure S3). The 2D images (single cell layer) were acquired using the Cytation 3 microscope. The original uncropped images are shown in Supplementary Figure S3, while the cropped regions presented in Figure 4 provide a zoomed-in view to better illustrate the effect of *Gelsemium* preparations on neurite outgrowth. Images were analyzed and quantified using the ImageJ Neurophology plugin (Figure 4 and Figure 5). The 2D view of neurite outgrowth of the SH-SY5Y cells allows the visualization of the formation of neurites and their projections between the cells (Figure 4 and Figure S3). After 2 weeks of chronic treatment with *Gelsemium* preparations, an increase in the neurite outgrowth parameters was observed (Figure 5). The 15C dilution was the most efficient dilution to increase the neurite count up to +109% when compared with the CTRL cells (Figure 4 and Figure 5A). In fact, compared with CTRL cells, 15C chronic treatment significantly improved the neurite length (up to 186% of increase, Figure 5B), number of attachment point (about 125% of increase, Figure 5C) as well as number of endpoints with 201.5% of improvements (Figure 5D). After two weeks of treatment, 9C significantly increased the neurite count (by up to 73.6%), the total neurite length (by approximately 86%), as well as the number of attachment points (by up to 90%) compared to CTRL cells (Figure 5A–C). Chronic treatment with 9C did not significantly increase the number of endpoints compared to CTRL cells, although a numerical increase of 104% was observed (*p* = 0.06; Figure 5D). Similarly, at the higher dilution 30C, *Gelsemium* significantly increased several neurite outgrowth parameters (up to 107%) compared to the CTRL group; however, the increase in endpoints did not reach statistical significance (*p* = 0.07; Figure 5D). Chronic treatment with the vehicle had no significant effect on neurite outgrowth in SH-SY5Y cells (Supplementary Figure S2).

### 2.6. Gelsemium Preparations Stimulate the Akt/mTOR Neurotrophic Pathway After Chronic Treatment

**Figure 6:**
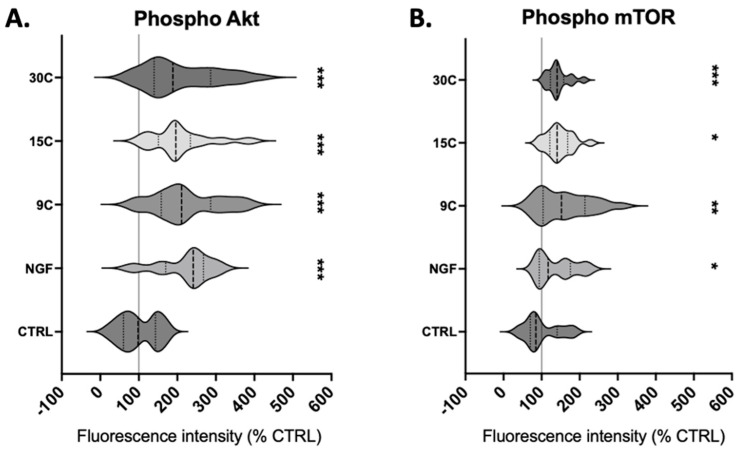
Activation of the Akt/mTOR signaling pathway following chronic exposure to *Gelsemium* preparations in human neuroblastoma cells (Table 5). SH-SY5Y neuroblastoma cells were treated for 2 weeks with *Gelsemium* preparations (9C, 15C, 30C) or NGF (positive control). Cells were lysed in MILLIPLEX^®^ MAP lysis buffer (Millipore Corp., Billerica, MA, USA) containing protease inhibitors. Equal amounts of total protein (20 μg per sample) were diluted in MILLIPLEX^®^ MAP assay buffer (Millipore Corp., Billerica, MA, USA) and analyzed according to the manufacturer’s protocol (overnight incubation at 4 °C). Phosphorylated Akt and mTOR levels were quantified using the Luminex^®^ 200 system (Millipore Corp.) and expressed as Fluorescence Intensity (measured by the Luminex^®^ system), normalized to 100% of untreated control (CTRL) cells (vertical grey line indicates normalization to CTRL). (**A**) Phosphorylated Akt levels in SH-SY5Y cells after chronic treatment with Gelsemium preparations (9C, 15C, 30C) or NGF. (**B**) Phosphorylated mTOR levels in SH-SY5Y cells after chronic treatment with Gelsemium preparations (9C, 15C, 30C) or NGF. Data are presented as violin plots showing the distribution of individual values per condition. The width of each violin reflects data density; the central dashed line indicates the median, and the dotted lines represent the first and third quartiles. Statistical analyses were performed on mean fluorescence intensity values, which are reported as mean ± SD in Table 5. Data were obtained from three independent biological experiments performed using different SH-SY5Y cell passages on different experimental dates. The reported *n* values (15–29 replicates per condition) correspond to the total number of technical replicates pooled across the three independent biological experiments. Statistics: One-way ANOVA followed by Dunnett’s multiple comparisons test. For (**A**): 9C/15C/30C vs. CTRL *** *p* < 0.001; NGF vs. CTRL *** *p* < 0.001; 9C/15C/30C vs. NGF: not significant. For (**B**): 9C/15C/30C vs. CTRL * *p* < 0.05, ** *p* < 0.01, *** *p* < 0.001; NGF vs. CTRL *** *p* < 0.001; 9C/15C/30C vs. NGF: not significant.

The Akt/mTOR signaling pathway plays a central role in regulating neuronal survival, growth, and plasticity through phosphorylation-dependent activation. To determine whether this pathway is modulated by *Gelsemium* preparations, we assessed the phosphorylation levels of Akt and mTOR in SH-SY5Y cells following a 2-week chronic treatment. Quantitative detection of phosphorylated Akt (Ser473) and mTOR (Ser2448) was performed using a Luminex assay; the results are presented as violin plots depicting the distribution of fluorescence intensity values (Figure 6).

*Gelsemium* 9C, 15C, and 30C significantly increased phospho-Akt (Ser473) levels by +122.9%, +107.4%, and +109.9%, respectively, compared with CTRL cells (Table 5, Figure 6A). The positive control NGF significantly ameliorated phospho-Akt (Ser473) levels by +121.9% compared to CTRL cells (Table 5, Figure 6A). Similarly, phospho-mTOR (Ser2448) levels increased by +58.6%, +46.4%, and +70.1% following treatment with 9C, 15C, and 30C, respectively, relative to CTRL cells. NGF also promoted mTOR phosphorylation, resulting in a +35.6% increase (Table 5, Figure 6B).

## 3. Discussion

**Figure 7:**
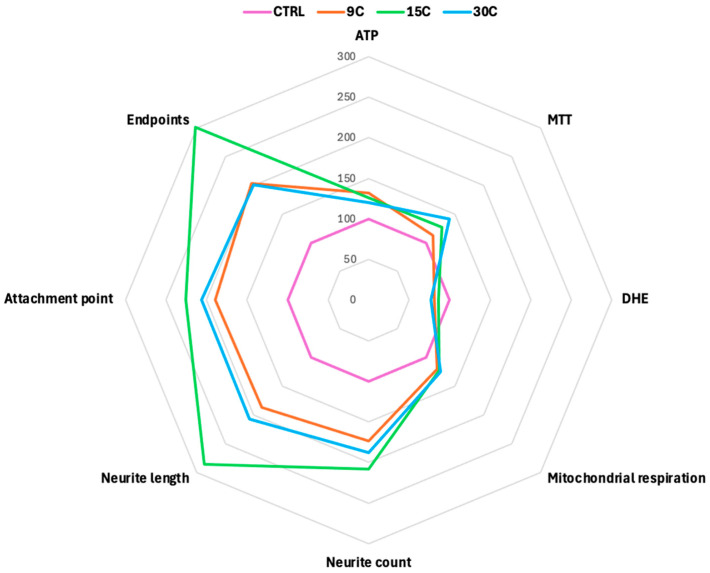
Chronic treatment with *Gelsemium* preparations (9C, 15C, 30C) was associated with differential changes in mitochondrial function and neurite outgrowth parameters in neuronal cells. Radar plot showing normalized values (CTRL = 100%, pink) for ATP content, cell viability (MTT), oxidative stress (DHE), mitochondrial respiration, and neurite extension parameters (neurite count, neurite length, attachment points, endpoints). Each *Gelsemium* preparation is represented by a distinct color (orange: 9C; green: 15C; blue: 30C). The results reveal that each dilution exerts specific and distinct effects on neuronal bioenergetics and morphology compared to control cells treated with the medium alone (pink), with variations observed across the different parameters. Overall, *Gelsemium* chronic exposure was associated with increases in energy metabolism and neuronal plasticity markers (ATP, mitochondrial respiration, neurite morphology) while reducing oxidative stress indicators (notably DHE across all dilutions).

In this study, we investigated the effects of chronic treatment with *Gelsemium* preparations at different dilutions 9C, 15C, and 30C on mitochondrial function, oxidative stress, cell viability, neurite outgrowth, and intracellular signaling in SH-SY5Y neuronal cells. The results indicate that prolonged exposure to these preparations is associated with coordinated changes in bioenergetic, redox, morphological, and signaling parameters under controlled in vitro conditions (Figure 7).

Previous experimental studies have reported that *Gelsemium* preparations can influence neuronal parameters, including mitochondrial activity, oxidative balance, and neurite outgrowth in cell models, as well as behavioral responses in animal studies following repeated administration. Thus, repeated administration of *Gelsemium* preparations (ranging from 4C to 30C) produced significant and reproducible anxiolytic-like effects in mice, particularly after several consecutive days of treatment [22,27]. These effects were observed following treatment durations of up to 8–9 days and were comparable to standard anxiolytic drugs such as diazepam or buspirone. Clinically, *Gelsemium* is commonly prescribed in repeated dosing regimens over several days for anxiety-related complaints [26]. Building on this background, we selected a 14-day exposure paradigm to investigate whether chronic treatment could elicit measurable cellular responses relevant to neuronal bioenergetics and plasticity. This approach was designed to characterize cumulative cellular effects rather than acute responses. One of the principal observations was a consistent increase across the tested dilutions in ATP production. These changes may reflect altered mitochondrial efficiency or increased metabolic activity, corroborated by parallel increases in oxygen consumption rate (OCR) and extracellular acidification rate (ECAR). Together, these results indicate that *Gelsemium* preparations are associated with increased oxidative phosphorylation and glycolysis, promoting a metabolically active cellular phenotype.

Mitochondrial function plays a central role in maintaining neuronal homeostasis, not only by supplying ATP through oxidative phosphorylation but also by regulating redox balance and signaling pathways involved in neuroplasticity [12]. Improved bioenergetics, as evidenced by enhanced OCR and ECAR, support the high energy demands of neurite extension and synaptic remodeling. Concurrently, the reduction in reactive oxygen species (ROS) levels, particularly superoxide anions, limits oxidative stress, which is known to impair cytoskeletal dynamics and synaptic integrity [28]. Our findings suggest that treatment with *Gelsemium* preparations is associated with a favorable metabolic and redox environment that is compatible with enhanced neurite outgrowth and neuronal plasticity, together with changes in energy-sensitive signaling pathways such as PI3K/Akt/mTOR.

Indeed, we observed a significant reduction in total superoxide anion levels. The observed reduction in total ROS may involve cytosolic antioxidant mechanisms or decreased overall superoxide generation rather than direct mitochondrial effects. Although the underlying mechanisms remain to be elucidated, this antioxidant effect may result from enhanced mitochondrial homeostasis or reduced oxidative burden due to improved bioenergetics. Given that mitochondrial oxidative stress is commonly reported in anxiety/depression and stress-related disorders, this redox modulation may be considered in the context of the broader literature in stress-related conditions [29,30].

Interestingly, differences were observed between ATP levels and MTT activity across dilutions. While ATP production tended to decrease across the tested dilutions, MTT activity was highest at 30C. Since the MTT assay primarily reflects cellular reductase activity and mitochondrial metabolic activity rather than cell viability per se, this apparent discrepancy may indicate differences in cellular metabolic state rather than direct effects on survival. The concomitant reduction in oxidative stress at 30C may also contribute to enhanced cellular resilience, consistent with a hormetic response model. Furthermore, no apoptosis- or caspase-based assays were performed in the present study; therefore, the observed changes in MTT activity should not be interpreted as direct evidence of altered cell survival.

More generally, the effects observed across the different *Gelsemium* dilutions were not consistently monotonic across all assays, as the strongest responses were associated with different dilutions depending on the biological parameter evaluated. Such non-monotonic response patterns have previously been described in biological systems and may be compatible with hormetic-like responses characterized by low-dose stimulation and non-linear dose–response relationships [31,32]. However, the present study was not designed to investigate the mechanisms underlying these dilution-specific effects, and therefore, no mechanistic conclusions can be drawn regarding the origin of these responses.

The dilutions investigated in the present study exceed Avogadro’s limit, and the physicochemical mechanisms potentially underlying the observed biological responses remain a matter of scientific debate. Several hypotheses, including nanoparticle-based mechanisms and water-structure-related models, have been proposed to explain biological responses to ultra-high dilutions. However, the present study was not designed to investigate these physicochemical aspects. Therefore, the observed effects should be interpreted as experimental observations at the cellular level rather than as evidence supporting a specific physicochemical mechanism.

Retinoic acid is known to promote neuronal differentiation and neurite extension in SH-SY5Y cells. In the present study, retinoic acid was applied after completion of the chronic treatment phase and identically to all experimental groups. Therefore, the observed differences in neurite morphology were detected following a common differentiation procedure and may reflect persistent effects of prior Gelsemium exposure on neuronal structural plasticity.

At the molecular level, *Gelsemium* preparations were associated with increased phosphorylation of Akt and mTOR, key components of the PI3K/Akt/mTOR pathway, which governs neuronal survival, growth, and synaptic plasticity [33]. Activation of this pathway represents a plausible mechanistic correlate of the observed effects. Considering that dysregulation of Akt/mTOR signaling has been implicated in anxiety and mood disorders [18,34], these findings support the hypothesis that *Gelsemium* exerts its effects, at least in part, through modulation of this signaling axis.

Several additional mechanisms may contribute to the observed effects. Previous studies have shown that *Gelsemium* preparations can influence neurosteroid synthesis and modulate gene expression patterns related to calcium signaling, inflammation, and cellular homeostasis in neuronal cells. Given the central role of calcium in mitochondrial regulation and ATP production, modulation of intracellular calcium dynamics represents a plausible avenue for future investigation [23,24].

Importantly, the convergence of mitochondrial, oxidative, morphological, and signaling readouts suggests a coordinated cellular response rather than an isolated or nonspecific effect [34]. In the context of this study, these combined changes are consistent with a neuroprotective-like cellular phenotype observed in vitro [35].

These findings are limited to a human neuroblastoma cell model and do not imply clinical efficacy. Rather, they provide experimental data describing how chronic exposure to *Gelsemium* preparations can influence cellular processes involved in neuronal energy metabolism and structural plasticity under controlled laboratory conditions [25].

While these observations are limited to an in vitro cellular model, they provide experimental evidence that chronic exposure to *Gelsemium* preparations can modulate cellular processes related to neuronal bioenergetics, redox balance, and structural plasticity under controlled laboratory conditions. Further studies using more physiologically relevant models will be required to determine the biological and translational relevance of these findings.

## 4. Materials and Methods

### 4.1. Chemicals and Reagents

Dulbecco’s Modified Eagle’s Medium (DMEM), fetal calf serum (FCS), penicillin/streptomycin, and dimethyl sulfoxide (DMSO) were purchased from Sigma-Aldrich (St. Louis, MO, USA). Glutamax was obtained from Gibco Invitrogen (Waltham, MA, USA). 4,5-Diaminofluorescein diacetate (DAF-2DA) was acquired from Chemodex (St. Gallen, Switzerland), and dihydroethidium (DHE) from Adipogen Life Sciences (Liestal, Switzerland). Nerve growth factor (NGF) was supplied by Lubio (Zürich, Switzerland).

### 4.2. Gelsemium Preparation Procedure

Dynamized dilutions of *Gelsemium sempervirens* (L.) Ait. f. were produced by Boiron (Messimy, France) in compliance with the European Pharmacopoeia (Ph. Eur., 11th Edition, 2022; preparation method 1.1.10 of monograph 2371) [36] and the French Pharmacopoeia (Fr. Ph., 11th Edition, 2013) [37] monograph for *Gelsemium*.

The starting material was a crude hydroalcoholic extract of dried underground parts of *Gelsemium sempervirens* (Herb’s International Service SARL, France; Batch H140503595), used to prepare the mother tincture as previously described [13,25]. *Gelsemium* is not listed as an endangered species by the International Union for Conservation of Nature (IUCN Red List of Threatened Species).

The first centesimal (1C) dilution was prepared by diluting the crude extract 1:100 in sterile water (OTEC, Aguettant, Lyon, France), followed by vigorous mechanical succussion using a potentizing machine. Subsequent serial 100-fold dilutions (9C, 15C, and 30C) were prepared according to the Hahnemannian centesimal dilution scheme, with intermediate dynamization at each step using the same solvent and procedure.

Sterile water processed identically through the same dilution and succussion steps was used as the vehicle control (placebo), as previously described [25].

### 4.3. Cell Culture Conditions and Treatment Paradigm

Human SH-SY5Y neuroblastoma cells were cultured in 10 cm dishes for 2 weeks. Every two days, half of the culture medium was replaced with fresh medium for all treatment groups. Cells were then treated with either 50 ng/mL nerve growth factor (NGF, positive control), *Gelsemium* preparations (9C, 15C, and 30C), vehicle (placebo), or medium alone (CTRL, control treated with the medium alone). Cells were passaged upon reaching approximately 80% confluence, and the medium was refreshed every other day throughout the treatment period to maintain the availability of growth factors and *Gelsemium* components.

Following the two-week treatment, cells from each condition were seeded into 96-well plates. After 48 h of culture, cells received a second 24 h treatment under the same conditions. Subsequently, analysis of the assays evaluating ATP content, cell viability (MTT), reactive oxygen species (ROS) levels, bioenergetic profiles, including oxygen consumption rate (OCR) and extracellular acidification rate (ECAR), neurite outgrowth, and associated signaling pathways was blindly performed. Characterization of vehicle chronic treatment of 14 days was performed on key experiments (Supplementary Figures S1 and S2). All experiments were repeated independently three times using different SH-SY5Y cell passages and performed on separate experimental dates.

### 4.4. ATP Levels Measurement

Total ATP content in SH-SY5Y cells was quantified using a bioluminescence assay (ViaLight™ HT, Cambrex Bio Science, Walkersville, MD, USA) following the manufacturer’s instructions, as previously described [25]. Cells were seeded in white 96-well plates at a density of 5 × 10^3^ cells per well. The assay is based on the luciferase-catalyzed reaction of ATP with luciferin, resulting in light emission proportional to ATP concentration. Luminescence was measured using the Cytation3 multi-mode plate reader (Biotek, Luzern, Switzerland).

### 4.5. MTT Assay for Assessing Cell Viability

Cell viability was evaluated using the MTT reduction assay following the protocol described by Wendt et al. [38]. SH-SY5Y cells were plated at a density of 5 × 10^3^ cells per well in 96-well plates and allowed to adhere overnight. Treatments were applied 48 h post-seeding. After treatment, cells were incubated with MTT reagent (3-(4,5-dimethylthiazol-2-yl)-2,5-diphenyltetrazolium bromide) prepared in DMEM for 3 h. Viable cells convert MTT into an insoluble violet formazan product via mitochondrial enzymatic activity. Following incubation, the formazan crystals were solubilized using DMSO, and the absorbance was measured at 550 nm using a Cytation3 multi-mode reader (BioTek). Absorbance values were expressed relative to untreated control cells, which were assigned a value of 100%.

### 4.6. Determination of Total Superoxide Anion Radical Levels, ROS Measurement

Following 10 days of chronic treatments, SH-SY5Y neuroblastoma cells were seeded into black 96-well plates at a density of 5 × 10^3^ cells per well for each treatment condition. After 48 h of culture, cells were subjected to a second 24 h treatment with *Gelsemium* preparations (9C, 15C, 30C), vehicle, or NGF. Subsequently, total superoxide levels were assessed using the fluorescent dye dihydroethidium (DHE, 10 μM for 20 min). Cells were incubated at room temperature in the dark on an orbital shaker. After two washes with HBSS (Sigma), fluorescence was measured using the Cytation3 Cell Imaging Multi-mode Reader. The formation of red fluorescent products was detected at 531 nm excitation and 595 nm emission. Fluorescence intensity directly reflected the total superoxide anion levels.

### 4.7. Mitochondrial Respiration and Glycolysis Measurement

The oxygen consumption rate (OCR) and extracellular acidification rate (ECAR), indicators of mitochondrial respiration and glycolysis, respectively, were measured using the Seahorse XF HS Mini Analyser (Agilent, Santa Clara, CA, USA), following the manufacturer’s recommendations. Cell culture microplates (Seahorse Bioscience, Agilent Technologies) were coated with 0.1% gelatin prior to use. After 13 days of chronic treatment in 10 cm dishes, SH-SY5Y cells were seeded into 8-well plates at a density of 12,000 cells/well in 80 μL of treatment medium containing 10% FCS, 1 g/L glucose, and 4 mM pyruvate, then incubated overnight. Prior to the assay, cells were washed with Phosphate-Buffered Saline (PBS) and incubated for 1 h at 37 °C in a CO_2_-free incubator with 200 μL of assay medium composed of DMEM without NaHCO_3_ and phenol red, supplemented with 18 mM glucose, 4 mM pyruvate, and 2 mM L-glutamine (pH 7.4). Basal OCR and ECAR measurements were recorded over a two-hour period using the XFp Analyzer (Agilent, Santa Clara, CA, USA). Bioenergetic profile of the cells after a chronic treatment of 14 days was established with the basal OCR and ECAR values.

### 4.8. Neurite Outgrowth Investigation: Cell Culture, Treatment, and Analysis

Cells were grown in T10 dishes and treated chronically until day 10 with either 50 ng/mL nerve growth factor (NGF, positive control), *Gelsemium* preparations 9C, 15C, and 30C, vehicle, or the medium alone (CTRL condition). Culture media were refreshed every two days, and cells were passaged when reaching 80% confluence to maintain optimal growth conditions. At day 10, cells were detached and seeded at a density of 5000 cells per well in a 2D surface culture by using collagen type I coated- black 96-well plates with clear bottoms (0.05 mg/mL) to promote adhesion and neurite extension. At day 11, neuronal differentiation was induced by culturing cells in medium supplemented with 10% fetal bovine serum and 10 μM retinoic acid (RA) for three days, to halt proliferation and promote neurite extension. Retinoic acid was applied after completion of the chronic treatment phase and identically to all experimental groups. Following differentiation, cells were fixed with 2% paraformaldehyde for immunostaining. Nuclei were labeled using DAPI (4′,6-diamidino-2-phenylindole, 0.5 µg/mL in PBS for 5 min at RT, fluorescence emission in the blue wavelength), while neurites were visualized with an anti-βIII-tubulin primary antibody followed by an Alexa Fluor 488-conjugated secondary antibody (fluorescence emission in the green wavelength). Imaging was performed directly in the 96-well plates using an automated high-content imaging system (Cytation3, Biotek). Quantitative analysis of neurite morphology—including neurite count, total length, branching points, and endpoints—was conducted using ImageJ software with the Neurphology plugin [19].

### 4.9. Neuroplasticity Pathway Characterization

To explore the molecular mechanisms underlying neurite outgrowth, protein expression levels of key neuroplasticity markers Akt and mTOR (including their phosphorylated forms) were assessed. Cells were grown in 10 cm dishes for 14 days, underwent differentiation, and the same chronic treatments and protocol as described above. After differentiation, cells were lysed, and protein levels were quantified using a targeted protein expression profiling approach based on Luminex technology with specific assay kits. This approach allowed for precise measurement of phosphorylated Akt and mTOR, providing insights into the activation of plasticity pathways associated with neurite outgrowth. Data were visualized as violin plots showing the median, first and third quartiles, and data distribution density. Group means ± SEM calculated from fluorescence intensity values measured by the Luminex^®^ system from independent experiments were used for statistical analyses (Table 5).

### 4.10. Statistical Analysis

Data are presented as mean ± standard error of the mean (SEM) and normalized to the control group (CTRL = 100%). Three independent biological experiments were performed using SH-SY5Y cells from different passages and conducted on different experimental dates. Each biological experiment included multiple technical replicates per treatment condition. Similar patterns were observed across the independent experiments. Raw data from the three independent experiments were pooled prior to normalization to the CTRL condition and subsequent statistical analysis. The n values reported in the figure legends correspond to the total number of technical replicates obtained across the three independent biological experiments. Statistical analyses were conducted using GraphPad Prism software 9 (version 9.3.1, San Diego, CA, USA. Data were analyzed using a one-way analysis of variance (ANOVA) followed by Dunnett’s post hoc multiple comparisons test. Two sets of post hoc analyses comparisons were performed:

(1) *Gelsemium*-treated conditions versus control (CTRL), to evaluate the effect of *Gelsemium* preparations on the measured parameters;
(2) *Gelsemium*-treated conditions versus positive control (NGF), to assess potential similar effects of the *Gelsemium* preparations compared to NGF.

Statistical significance was considered at *p* < 0.05.

Figures are presented as mean ± SEM to facilitate visualization of differences between group means, whereas tables report mean ± SD to provide information on the variability and dispersion of the underlying data.

## 5. Conclusions

Altogether, our results show that chronic treatment with *Gelsemium* preparations is associated with measurable changes in bioenergetic, antioxidant, and neurotrophic parameters in neuronal cells, including increased Akt/mTOR signaling. These effects are reflected in the modulation of mitochondrial bioenergetics and cellular metabolism, including ATP production, mitochondrial membrane potential, oxidative metabolism, mitochondrial respiration, glycolysis, and neurite outgrowth. These findings extend the existing experimental literature on *Gelsemium* by providing cellular-level insights into its bioenergetic and metabolic effects. These findings extend the existing experimental literature on *Gelsemium* by providing cellular-level insights into its bioenergetic, metabolic, and signaling effects in neuronal cells under in vitro conditions. Further studies using primary neuronal cultures, stress paradigms, and in vivo models will be required to determine the physiological and translational relevance of these observations.

## Figures and Tables

**Table 1:** The data are presented as mean ± SD corresponding to the number of replicates (*n*) (Figure 1). Values are normalized on 100% of the control group (CTRL treated with the medium alone).

	CTRL (*n*)	NGF (*n*)	9C (*n*)	15C (*n*)	30C (*n*)	*p* Value (One-Way ANOVA)
ATP assay	100 ± 24.3 (17)	126 ± 13.4 (20)	131.9 ± 11.6 (24)	126.1 ± 10.4 (24)	119.9 ± 12.9 (29)	0.001
MTT assay	100 ± 17.7 (25)	138.7 ± 42.7 (17)	112.6 ± 18.9 (19)	127.4 ± 20.6 (17)	141.2 ± 52.7 (33)	0.001

**Table 2:** The data are presented as mean ± SD corresponding to the number of replicates (*n*) (Figure 2). Values are normalized on 100% of the control group (CTRL treated with the medium alone).

	CTRL (*n*)	NGF (*n*)	9C (*n*)	15C (*n*)	30C (*n*)	*p* Value (One-Way ANOVA)
DHE assay	100 ± 8.8 (18)	74.4 ± 7.1 (17)	80.9 ± 7.9 (15)	85.9 ± 9.2 (14)	76.9 ± 13.3 (17)	0.001

**Table 3:** Data are presented as mean ± SD corresponding to the number of replicates (*n*) shown in Figure 3. Values were normalized to 100% of the control group (CTRL, cells treated with medium alone).

	CTRL (*n*)	NGF (*n*)	9C (*n*)	15C (*n*)	30C (*n*)	*p* Value (One-Way ANOVA)
OCR	100 ± 5.068 (48)	128.9 ± 24.51 (48)	119.7 ± 20.27 (48)	123.1 ± 20.20 (48)	125.4 ± 33.40 (40)	0.001
ECAR	100 ± 3.859 (48)	126.7 ± 32.96 (48)	130.9 ± 35.21 (48)	119 ± 16.24 (37)	125.8 ± 42.6 (48)	0.001
ECAR/OCR ratio *	100 ± 0.00 (12)	98.22 ± 19.99 (12)	108.8 ± 18.99 (12)	95.78 ± 13.41 (10)	108.0 ± 11.22 (10)	0.03

* For the ECAR/OCR ratio, values were calculated from the mean OCR and ECAR values obtained in each independent biological experiment and are presented as mean ± SD. The reported *n* values correspond to the number of independent biological experiments (CTRL, NGF, and 9C: *n* = 12; 15C and 30C: *n* = 10). Statistical analysis was performed using a mixed-effects model (REML).

**Table 4:** Data are presented as mean ± SD corresponding to the number of cells (*n*) (as shown in Figure 5). Values are normalized on 100% of the control group (CTRL treated with the medium alone).

2-D Collagen	CTRL (*n* = 1289)	NGF (*n* = 2062)	9C (*n* = 2338)	15C (*n* = 2363)	30C (*n* = 2280)	*p* Value (One-Way ANOVA)
Number of neurites	100 ± 44.1	209.1 ± 101.6	173.6 ± 68.4	208.5 ± 109.2	187.6 ± 60.4	0.001
Total neurite length	100 ± 45.3	265.6 ± 178.5	186.4 ± 84.6	286.3 ± 224.2	207.6 ± 93.4	0.001
Attachment points	100 ± 42.6	233.1 ± 71.9	189.5 ± 53.3	225 ± 66	206.3 ± 50.2	0.001
Endpoint	100 ± 42.7	265.1 ± 218.8	203.6 ± 81.8	301.5 ± 263.6	200.5 ± 115.3	0.001

**Table 5:** The data are presented as mean ± SD of fluorescence intensity values corresponding to the number of replicates (*n*) (as shown in Figure 6). Values are normalized on 100% of the control group (CTRL treated with the medium alone).

	CTRL (*n*)	NGF (*n*)	9C (*n*)	15C (*n*)	30C (*n*)	*p* Value (One-Way ANOVA)
Phospho Akt	100 ± 49.5 (15)	221.9 ± 67.5 (17)	222.9 ± 85.5 (23)	207.4 ± 75.7 (27)	209.9 ± 95.3 (29)	0.001
Phospho mTOR	100 ± 45.4 (26)	138.5 ± 49.2 (27)	158.6 ± 68.8 (22)	146.4 ± 34.3 (18)	170.1 ± 67.6 (19)	0.001

## Data Availability

The datasets used and/or analysed during the current study are available from the corresponding author on reasonable request.

## Supplementary Materials

The following supporting information can be downloaded at: https://www.mdpi.com/article/10.3390/ijms27125409/s1.

## Abbreviations

The following abbreviations are used in this manuscript:

ATP	Adenosine triphosphate
C	Centesimal dilution
CTRL	Control
DAF-2DA 4	4,5-Diaminofluorescein diacetate
DMEM	Dulbecco’s Modified Eagle’s Medium
DMSO	Dimethyl sulfoxide
DHE	Dihydroethidium
ECAR	Extracellular acidification rate
FCS	Fetal calf serum
GABA	Gamma-aminobutyric acid
HBSS	Hank’s Balanced Salt Solution
IUCN	International Union for Conservation of Nature
M	Mean
mTOR	Mammalian target of rapamycin
MDD	Major Depressive Disorder
MTT	3-(4,5-Dimethylthiazol-2-yl)-2,5-diphenyltetrazolium bromide
NGF	Nerve Growth Factor
OCR	Oxygen consumption rate
OXPHOS	Oxidative phosphorylation
PBS	Phosphate-buffered saline
PI3K	Phosphatidylinositol 3-kinase
Akt	Protein kinase B
ROS	Reactive oxygen species
RA	Retinoic acid
RT	Room temperature
XFp	Seahorse Extracellular Flux XFp Analyzer
SD	Standard deviation
SEM	Standard error of the mean
