# Supplementary material for: Chronic Exposure to Gelsemium Preparations Alters Mitochondrial Bioenergetics, Neurite Outgrowth, and Akt/mTOR Signaling in Human Neuronal Cells
Source: Int J Mol Sci. 2026 Jun 16;27(12):5409. doi: 10.3390/ijms27125409 (PMC13300635; doi:10.3390/ijms27125409)

Supplementary Figure S1: Characterization of vehicle chronic treatment on human neuroblastoma cells across different assays. Values represent the mean ± SEM of three independent experiments (A. (ATP assay): 9-17 replicates per condition, B. (MTT assay): 16-25 replicates per condition, C. (Basal mitochondrial respiration): 36-48 replicates per condition, D. (Basal glycolysis): 32-48 replicates per condition, E. (DHE assay): 17-18 replicates per condition), normalized to 100% of untreated control (CTRL) cells. Statistical analysis was performed using unpaired Student's t-test versus CTRL: ns= not significant.

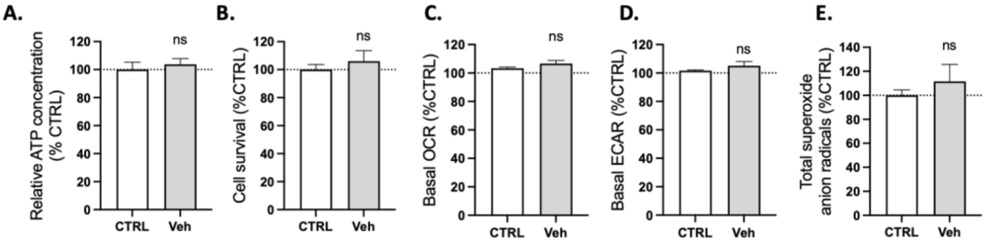

Supplementary Figure S2: Characterization of chronic vehicle treatment on neurite outgrowth parameters in human neuroblastoma cells. (A) Representative fluorescence images of SH-SY5Y cells after chronic vehicle (Veh) or untreated control (CTRL) exposure (green: β3-tubulin; blue: nuclei, DAPI). Scale bar: 100 μm. Quantification of neurite outgrowth parameters, including neurite count (B), neurite length (C), attachment points (D), and endpoints (E), expressed as a percentage of CTRL. Values represent the mean ± SEM of three independent experiments (number of analyzed cells: CTRL, N = 1289; Veh, N = 861), normalized to 100% of untreated control (CTRL) cells. Statistical analysis was performed using an unpaired Student's t-test versus CTRL: ns= not significant.

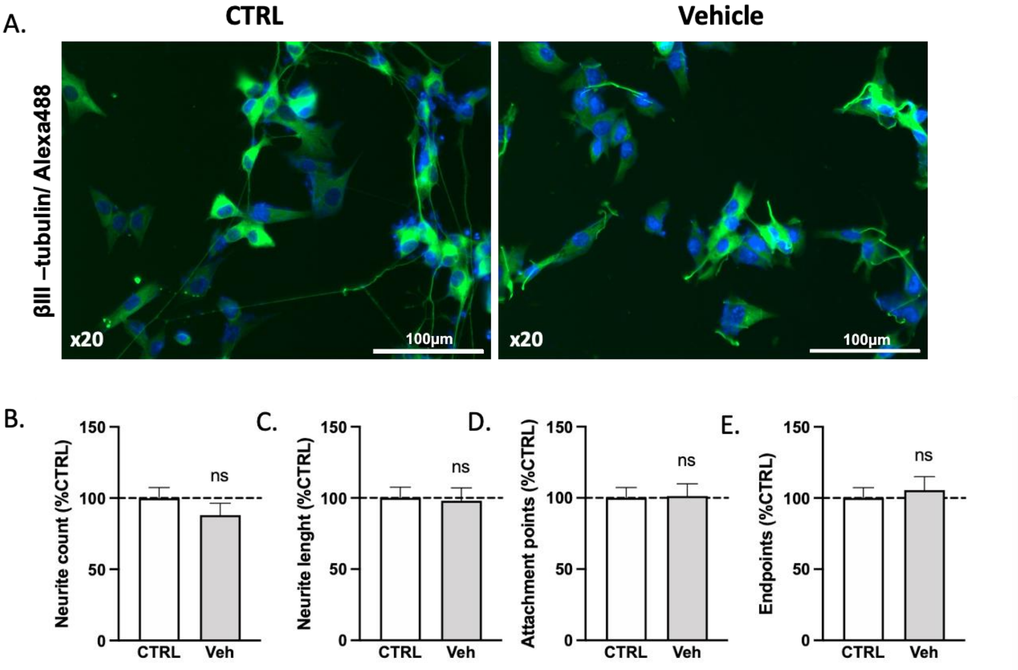

Supplementary Figure S3: Original uncropped images corresponding to the representative fields.

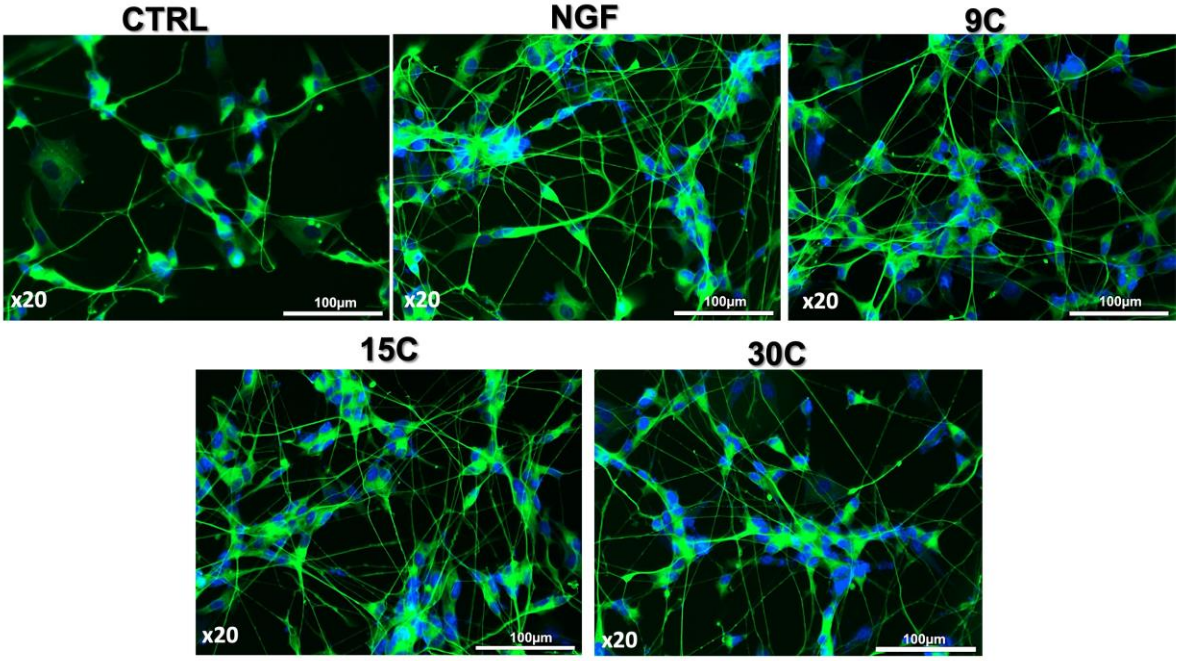

Supplement: Supplementary file 1 [file ijms-27-05409-s001.zip › ijms-4347964-supplementary.pdf]
